# Aged female and male C57BL/6J mice have reduced alcohol self-administration and altered acute alcohol withdrawal compared to younger animals

**DOI:** 10.3389/fnagi.2026.1792513

**Published:** 2026-03-19

**Authors:** Douglas B. Matthews, Aidan Riley, Lydia Staebell, Jared Kendrick, Jadyn Hartwig, Samantha Feller, Pravesh Sharma

**Affiliations:** 1 Department of Psychology, University of Wisconsin – Eau Claire, Eau Claire, WI, United States; 2 Mayo Clinic Health System, Eau Claire, WI, United States

**Keywords:** aging, alcohol, alcohol withdrawal, drinking in the dark (DID), lifespan

## Abstract

**Introduction:**

As the age of the world’s population continues to increase it is important to investigate behaviors, such as alcohol consumption, that may negatively impact the health of the older population.

**Materials:**

In the present study, aged, young adult and adolescent female and male C57BL/6J mice underwent a measure of acute alcohol withdrawal via a handling induced convulsion study followed by a drinking in the dark procedure to measure alcohol self-administration.

**Results:**

We report that aged animals enter acute alcohol withdrawal later than younger animals as measured by later onset of handling induced convulsions. The later onset is likely due to reduced alcohol metabolism. Furthermore, aged animals consumed significantly less alcohol in the drinking in the dark paradigm but had similar blood alcohol concentrations compared to younger animals.

**Discussion:**

The current work demonstrates for the first time differential alcohol self-administration and acute alcohol withdrawal in aged animals compared to younger animals.

## Introduction

The age of the population is dramatically increasing in most countries. For example, the number of people over the age of 60 is predicted to double to 2.1 billion people in the 20-year time frame between 2030 to 2050 (World Health Organization, Ageing and Health, 2025). Similarly, the percent of the population in the United States that is over the age of 65 years increased from 12.4% in 2004 to 18% in 2024 (The United States Census Bureau, 2025). Older adults use healthcare significantly more than younger adults (Lassman et al., 2014; Jones and Dolsten, 2024) and the significant increase in the population age threatens to overwhelm healthcare systems (Tampi et al., 2015; Jones and Dolsten, 2024), a result often referred to as a silver tsunami. Understanding behaviors that can negatively impact the health of the aged population is a critical public health concern.

Older adults continue to consume alcohol, and recent epidemiological data shows that alcohol consumption rates are increasing in the aged population (Calvo et al., 2020; Tampi et al., 2022; Keyes, 2023; White et al., 2023). Alcohol consumption in older people can be a significant health concern as alcohol drinking has been linked to increases in the risk of several types of cancer (Islami et al., 2024; Esser et al., 2024; Dorobisz et al., 2025), injury, brain bleeds and death due to falling (Zirulnik et al., 2024) and is a contributing factor to the development of Alzheimer’s disease and related dementia (for review see Anton et al., 2025; Zahr, 2024; Chang et al., 2025). Understanding the effect of alcohol in older demographics is necessary to reduce risk factors in this rapidly growing segment of the population (Matthews and Koob, 2023).

Due to ethical concerns, animal models are often used to investigate the effect of alcohol in aged subjects (Matthews and Rossmann, 2023). Comparing aged rodents to younger subjects, alcohol exposure results in aged subjects being more sensitive to the ataxic (Van Skike et al., 2010; Novier et al., 2013; Matthews and Mittleman, 2017), hypnotic (Ornelas et al., 2015; Gano et al., 2017; Perkins et al., 2018), spatial memory (Novier et al., 2013; Matthews et al., 2020; Anton et al., 2024), and hypothermic (Watson et al., 2020) effects of alcohol. In addition, recent work has revealed sex-dependent effects of acute alcohol exposure in aged animals compared to younger animals. For example, aged female rats have a greater anxiolytic response to acute alcohol than aged male rats, but aged female rats also have a reduced motoric activation to alcohol compared to aged male rats (Matthews et al., 2024). Furthermore, aged female rats have a reduced ataxic effect to acute alcohol compared to aged males (Kerr et al., 2025) and aged females have less loss of righting reflex (LORR) compared to aged males (Perkins et al., 2018). Given recent data also has shown that older females are increasing their alcohol consumption (McKetta and Keyes, 2020; for review see White, 2020), it is important to investigate sex-dependent effects of alcohol in aged subjects (Matthews et al., 2024; McKee et al., 2025; Witkiewitz and Leggio, 2025).

Understanding the impact that advanced age and sex have on alcohol self-administration has been understudied in animal models. Furthermore, general measures of acute alcohol withdrawal have not been rigorously investigated in relation to aged female and male animal subjects. Mouse models are often used to study both alcohol consumption and acute alcohol withdrawal. For example, C57BL/6J (B6) mice readily consume alcohol in a variety of procedures including 24-h two bottle self-administration, limited access, and operant responding (Faccidomo et al., 2025; Yoon et al., 2025; reviewed in Radke et al., 2021). Furthermore, B6 mice will consume sufficient alcohol in a 2-h Drinking-in-the-Dark (DID) self-administration session to reach levels of an alcohol binge, ~80 mg/dL blood alcohol level (Rhodes et al., 2005; Thiele and Navarro, 2014; Bauer et al., 2022). In addition, acute alcohol withdrawal can be assessed in B6 mice by determining changes in handling induced convulsions (HIC) following an alcohol challenge (Buck et al., 1997; Philip et al., 2010). However, alcohol self-administration and acute alcohol withdrawal are understudied in aged female and male mice compared to younger subjects.

The current project investigated acute alcohol withdrawal via HIC in aged, adult and adolescent female and male B6 mice. In addition, we determined alcohol self-administration in the same animals 6 weeks later using a 4-day drinking-in-the-dark paradigm. We report that while total HIC is similar in females and males across the three ages, the temporal expression of HIC is different in aged animals compared to younger animals, particularly aged female subjects, likely due to difference in alcohol metabolism. Furthermore, we demonstrate that baseline HIC increases as a function of age. Finally, we report that alcohol self-administration is significantly less in aged animals compared to younger animals although resultant blood alcohol levels appear similar.

### Methods

*Subjects*: A total of 92 C57BL/6J mice (*n* = 46 males and 46 females) of three ages (adolescent *n* = 28 [5 to 6 weeks]; young adult *n* = 32 [14 weeks]; aged *n* = 32 [78 weeks] at the time of arrival) were used in the current project. All subjects were purchased from Jackson Laboratory and housed in an approved animal colony at the University of Wisconsin – Eau Claire. All animal procedures were approved by the UW-Eau Claire IACUC. Animals were housed 2 to 5 per cage with same age and sex matched subjects except during the drinking in the dark experiment (see below). Subjects always had free access to food (Teklad #2018, 18% protein) and tap water except for 2 h during the drinking in the dark experiment (see below).

*Handling induced convulsions*: One week following arrival in the colony 62 subjects (adolescent male and female *n* = 9 per sex; adult male and female, aged male and female *n* = 11 per age and sex) underwent an assessment of acute alcohol withdrawal via HIC as previously described (Buck et al., 1997; Metten and Crabbe, 2005). Briefly, a baseline HIC score was determined for each animal prior to being weighed, and then subjects were injected intraperitoneally with 4.0 g/kg alcohol (20% v/v) before being placed back into their home cage. HIC was scored every hour post alcohol injection for the next 10-h. For each HIC score following alcohol exposure, baseline HIC (pre-alcohol) was subtracted to determine a withdrawal score due to previous alcohol administration. Furthermore, if the calculated HIC score was negative (i.e., a greater baseline HIC score than a post alcohol HIC score) a value of zero was recorded. Total HIC score was the summed HIC scores from 1 to 10 h post alcohol injection.

*Drinking in the Dark*: Six weeks following the HIC procedure, alcohol self-administration was assessed via a 2-h Drinking in the Dark (DID) assay over four subsequent days. All animals from the HIC procedure, except 20 animals (*n* = 4 for adult female and male and aged female and male and n = 2 for adolescent female and male), were used in the DID study. The removal of 16 animals from the DID protocol was due to an unexpected construction project in a room adjacent to the colony thereby disrupting experiments while 4 animals were removed for health reasons prior to the start of the DID project. Three days prior to the DID study, animals were individually housed with free access to food and water. The day before the DID study, subjects were weighed and this bodyweight was used to determine alcohol consumption for each day during the DID procedure. Two hours following lights off (Rhodes et al., 2005), water bottles were removed, and 15 mL drinking tubes fitted with stoppers (Amuza Inc., San Diego CA) and filled with 20% alcohol (v/v) were placed on the cages. The amount of alcohol in the drinking tube prior to placement on the cage and 2-h later were recorded. In addition, a similarly treated drinking tube was placed on an empty cage as a measure of fluid leakage. All work was done under red lights to minimize circadian disruption. Amount of fluid consumed was determined by subtracting the final fluid amount from the initial fluid amount and then reducing the consumption amount for each subject by any fluid leaking from the leak tube for each individual day. Finally, based on the previously determined body weight, g/kg alcohol was determined. In a subset of subjects (n = 2 for adolescent female and male mice and *n* = 3 for adult and aged female and male mice), immediately following determination of fluid consumed, animals were moved to a new experimental room, rapidly decapitated and trunk blood collected in heparinized capillary tubes for blood alcohol analysis (see below).

*Blood alcohol determination*: Due to HIC analysis suggesting an age-dependent effect of acute alcohol withdrawal that was dependent on the time following alcohol injection, 30 subjects (*n* = 5 per sex and age) were administered 4.0 g/kg alcohol (20%, intraperitoneally) and 4 h later (the time when HIC scores first began to be positive) subjects were rapidly decapitated and trunk blood collected in heparinized capillary tubes. The blood was centrifuged to separate plasma, and BAC was determined via an Analox AM-1(Analox Instruments, Suite 3, Faraday House, UK) per manufactories specifications.

*Statistical analysis*: Data were analyzed via ANOVA with appropriate post-hoc tests to determine if age and sex impacted acute alcohol withdrawal or alcohol self-administration. In addition, we used Pearson correlation to investigate the relationship between alcohol consumption and subsequent blood alcohol concentrations.

## Results

### Acute alcohol withdrawal

**Figure 1:**
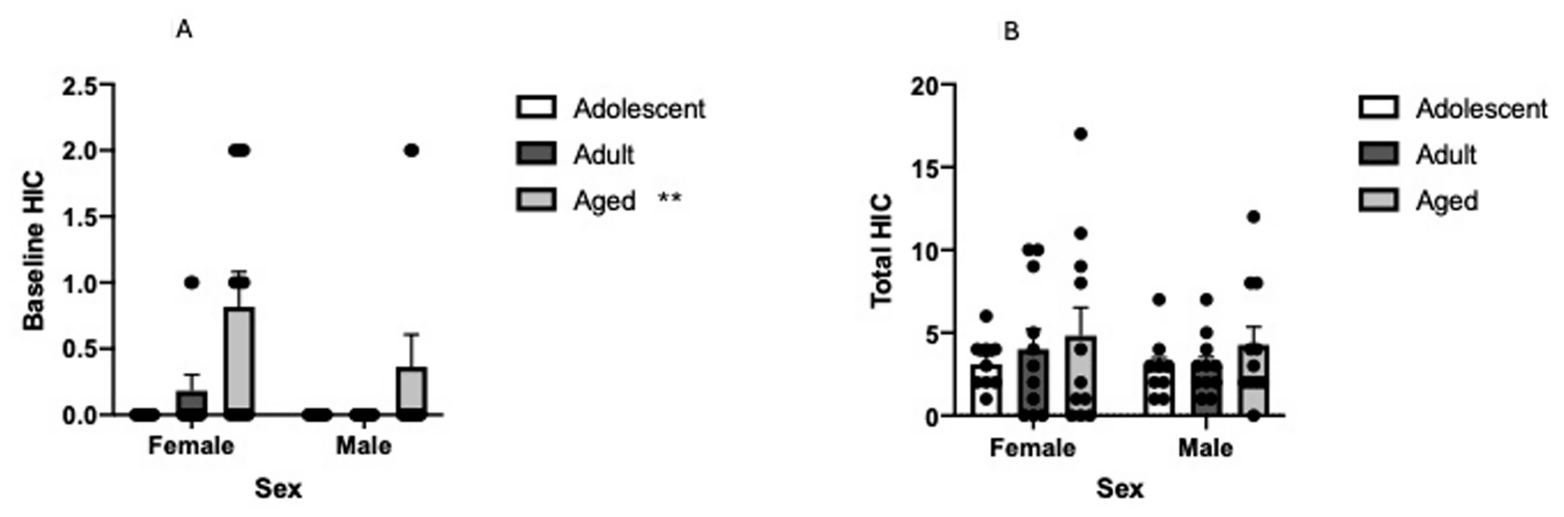
**(A)** Aging significantly increases baseline, pre-alcohol, handling induced convulsions in aged B6 mice. Mean baseline HIC in female and male aged, young adult and adolescent B6 mice. Error bars denote SEM, ** *p* < 0.01. **(B)** Total handling induced convulsion following 4.0 g/kg alcohol exposure was not impacted by age or sex in B6 mice. Mean handling induced convulsion scores summed over the 10 h post alcohol test period. Error Bars denote SEM.

We first investigated if age and sex of subjects significantly impacted baseline (pre-alcohol administration) handling induced convulsions and found that age of the animal significantly effected baseline HIC scores (Two Way ANOVA, age [3] X sex [2], main effect of age, *F* = 7.496, df(2,56), *p* = 0.0013). Sidak’s *post hoc* comparisons revealed that aged animals, regardless of sex, had significantly higher baseline HIC scores compared to adolescent (t = 3.498, df(56), *p* = 0.0028) or adult animals (t = 3.12, df(56), *p* = 0.0085). See Figure 1A. We next investigated if HIC due to acute alcohol withdrawal was different as a factor of sex and age and found that total HIC was not significantly impacted by the age of the subject or the sex of the subject (Two Way ANOVA, age [3] X sex [2], no significant interaction or main effects). See Figure 1B.

**Figure 2:**
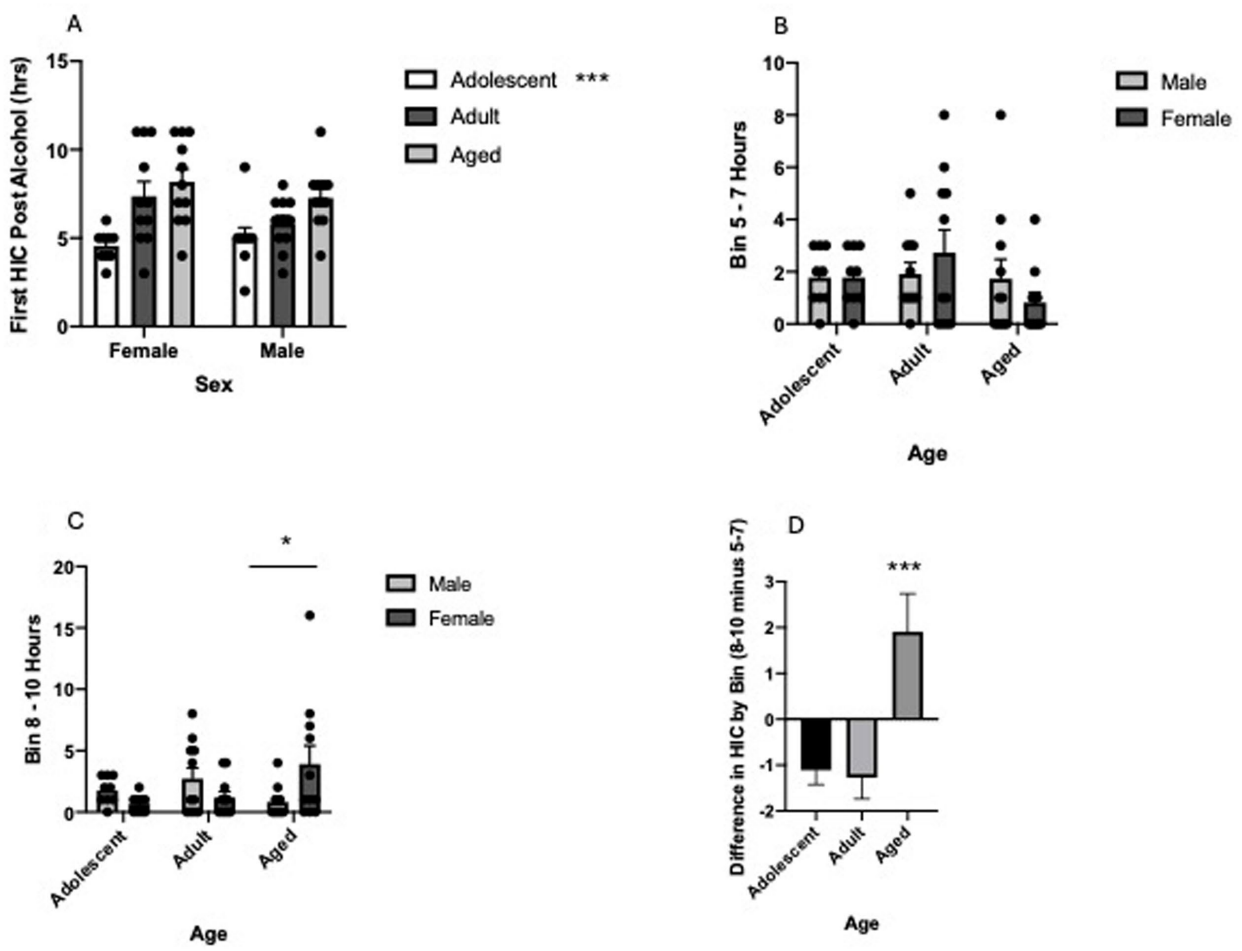
**(A)** Older B6 mice have first positive handling induced convulsion scores significantly later than adolescent B6 mice regardless of sex. Mean hour following the 4.0 g/kg alcohol injection when subjects first had a positive (post alcohol HIC greater than pre alcohol HIC) score. Error bars denote SEM, ***p < 0.001. **(B)** No significant difference in handling induced convulsions in the post alcohol 5 h to 7 h time frame. Mean HIC score in subjects in the 5 to 7 h time bin following the 4.0 g/kg alcohol administration. Error bars denote SEM. **(C)** Significant sex-dependent effect in handling induced convulsions in the post alcohol 8 h to 10 h time frame. Mean HIC score in subjects in the 5 to 7 h time bin following the 4.0 g/kg alcohol administration. The significant interaction is driven primarily by a significant difference in HIC in aged female compared to aged male subjects. Error bars denote SEM, *p < 0.05. **(D)** Significant effect of handling induced convulsion score based on time following alcohol inject is aged-dependent. Mean difference HIC score when the 5 to 7 h post alcohol score is subtracted from the 8 to 10 h post alcohol score. A negative score indicates greater HIC scores in the 5 to 7 h post alcohol time frame while a positive score indicates greater HIC scores in the 8 to 10 h post alcohol time frame. Error bars denote SEM, ***p < 0.001.

We and others have previously demonstrated that aged animals metabolize alcohol slower following administration of a high dose of alcohol (greater than ~ 3.0 g/kg, i.p.; Perkins et al., 2018; Watson et al., 2020), and consequently it is possible that age of the subject impacts the onset of alcohol withdrawal and subsequent elevated HIC scores following the 4.0 g/kg alcohol administration. To investigate this, we determined the 1-h time bin post alcohol administration when subjects first had a positive HIC score (post alcohol HIC greater than baseline HIC). In this case we found that age once again significantly impacted the onset of the first positive HIC (Two Way ANOVA, age[3] by sex[2], *F* = 11.12, df(2,56), *p* < 0.0001). Sidak’s post hoc comparisons revealed that adolescent animals had significantly earlier HIC onset compared to adult (t = 2.89, df(56), *p* = 0.0163) and aged subjects (t = 4.701, df(56), *p* < 0.0001). See Figure 2A. To further investigate the differential temporal onset of acute alcohol withdrawal following a 4.0 g/kg alcohol challenge we divided a portion of the 10-h post alcohol administration period into two-time bins of 3 h. Specifically, we calculated cumulative HIC scores in the 5–7-h post alcohol administration time bin and the 8–10-h post alcohol administration time bin and compared them as a function of age and sex of subject. We did not use the 1–4-h post alcohol administration time bin because the intoxication level produced by the 4.0 g/kg alcohol injection led to almost no HIC scores. In support of the analysis of the first bin with a positive HIC score, a significant 3-way interaction was found when investigating the impact of age and sex on the two HIC 3-h time bins (Three-Way ANOVA with repeated measures, age [3] X sex [2] X time bin [2], three way interaction of age, sex and time bin, *F* = 7.969, df(2,56), *p* = 0.0009). To better understand this interactive effect and focus on age and sex of subjects, we conducted follow-up analysis for both the 5–7-h time bin and the 8–10-h time bin. No significant effect of age of subject or sex of subject was found in the 5–7-h time bin (Two Way ANOVA, age [3] X sex [2], no significant interaction or main effect, all p’s > 0.05) but a significant interaction of age and sex was found in the 8–10-h time bin (Two Way ANOVA, age [3] X sex [2], significant interaction of age and sex, *F* = 5.085, df(2,56), *p* = 0.0093). Sidak’s multiple comparisons test revealed that the significant interaction was driven by a significant sex difference in aged animals (t = 2.754, df(56), *p* = 0.0236). See Figures 2B,C. Finally, we calculated a HIC difference score by subtracting HIC in the 5–7 h-bin from the 8–10-h bin and analyzed the resultant data as a function of age. In this analysis, a positive difference score demonstrates greater HIC in the 8–10-h time bin while a negative difference score demonstrates a greater HIC in the 5–7-h time bin. As expected from the previous analysis, age of the subject significantly impacted the difference score (One Way ANOVA, main effect of age, *F* = 9.093, df(2,59), *p* = 0.0004). Dunnett’s *post hoc* analysis revealed that aged animals had significantly different HIC scores as a function of time compared to both adolescent (q = 3.456, df(59), *p* = 0.002) and adult subjects (q = 3.838, df(59), *p* = 0.0006) indicating aged animals had significantly later HIC scores compared to younger animals. See Figure 2D.

**Figure 3:**
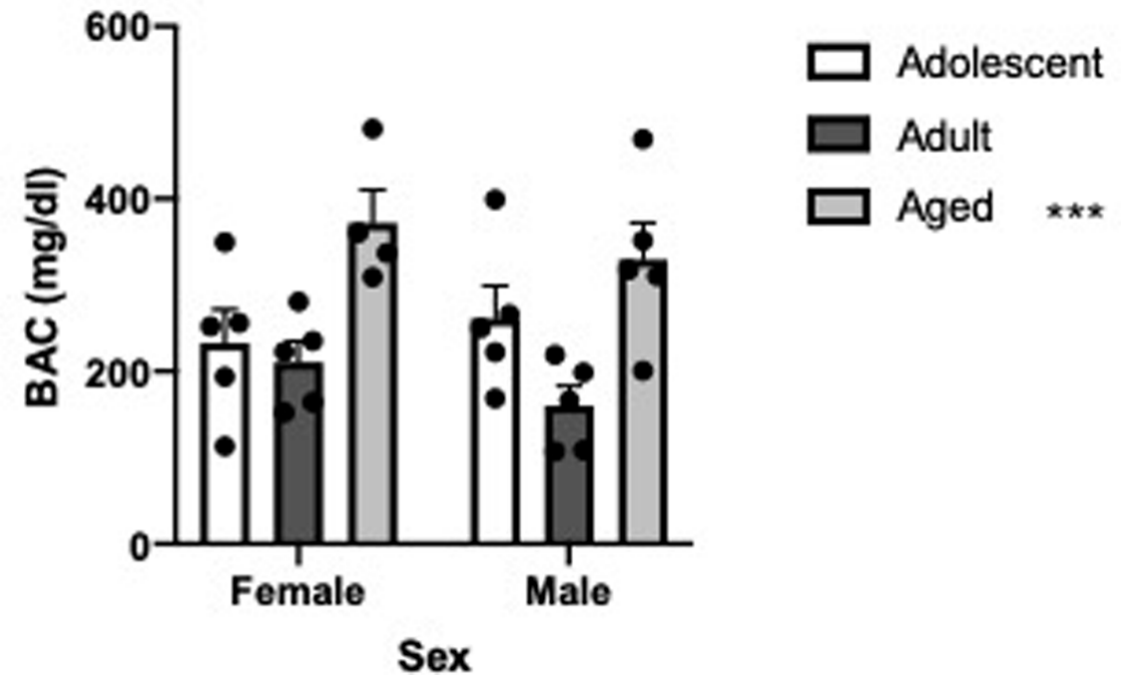
Blood alcohol concentrations are significantly elevated in aged B6 mice 4 h following a 4.0 g/kg alcohol injection. Mean blood alcohol concentration (mg/dL) with error bars denoting SEM. ***p < 0.0001.

The later onset of HIC in aged animals suggests that the aged subjects are metabolizing alcohol slower and consequently entering alcohol withdrawal significantly later than younger animals. To investigate this, we injected a novel group of aged, adult and adolescent female and male animals with 4.0 g/kg alcohol and 4 h later collected trunk blood for BAC determination. In support of the conclusion that aged animals are entering acute alcohol withdrawal later than younger animals we found differential blood alcohol concentrations (Two Way ANOVA, age [3] X sex [2], main effect of age, *F* = 11.07, df(2,23), *p* = 0.0004). Sidak’s multiple comparison post test revealed that aged animals had significantly higher BAC compared to both adolescent animals (t = 2.934, df(23), *p* = 0.0222) and adult animals (t = 4.674, df(23), *p* = 0.0003). See Figure 3.

### Drinking in the dark

**Figure 4:**
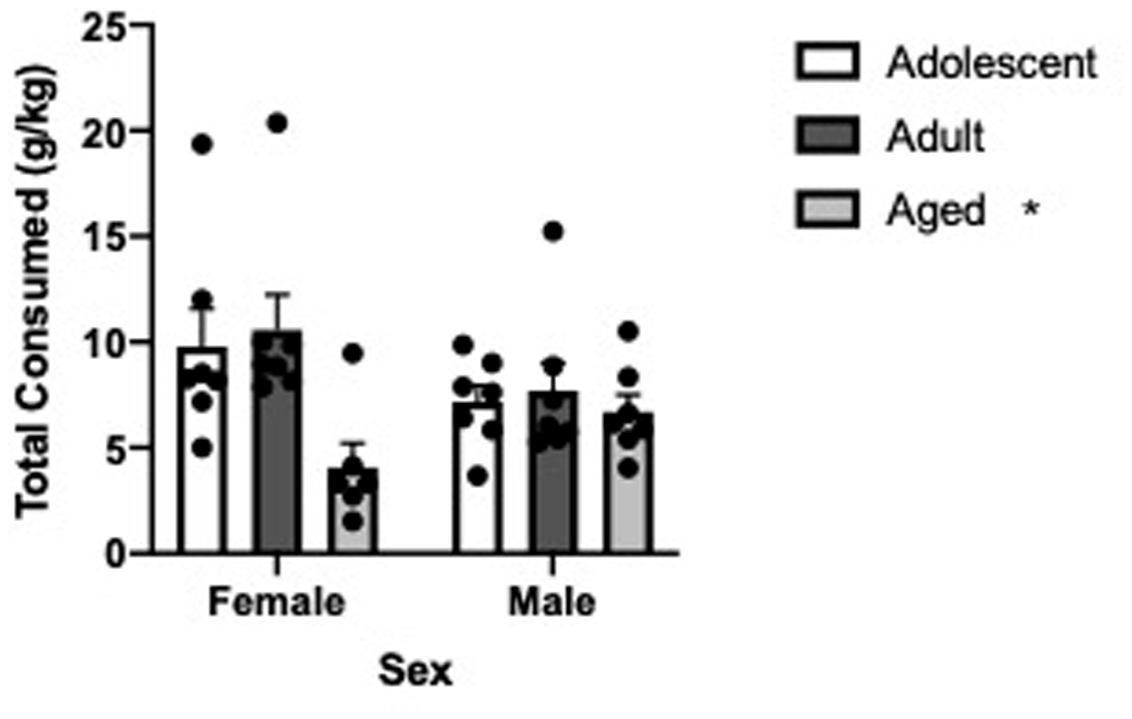
Mean cumulative alcohol consumption during the 4 day drinking in the dark paradigm is significantly less in aged B6 animals compared to younger subjects. Error bars denote SEM. * *p* < 0.05.

To investigate how age and sex impacted a short term DID procedure, we first summed drinking across the 4-days to determine total alcohol consumption and analyzed consumption using age and sex as factors. In this analysis, age significantly decreased alcohol consumption in the DID experiment (Two Way ANOVA, age [3] X sex [2], main effect of age, *F* = 4.47, df(2,35), *p* = 0.0187). Sidak’s multiple comparison test revealed that aged animals drank significantly less than adult animals (t = 2.813, df(35), *p* = 0.0238) and tended strongly to drink less than adolescent animals (t = 2.328, df(35), *p* = 0.0755). See Figure 4. To investigate the relationship between alcohol consumed on day 4 of the DID procedure and blood alcohol concentrations, we collected trunk blood on a small sample of animals. As expected, a significant positive correlation was found between amount consumed and underlying BAC in subjects (r^2^ = 0.4124, *p* = 0.0133). Finally, due to small sample sizes, we collapsed BAC data across sex and investigated if differential BAC following the final 2-h DID procedure was different as a function of age and found no significant difference in blood alcohol concentration in the three ages (One way ANOVA, *F* = 1.027, df(2,11) *p* = 0.3902).

## Discussion

We report, to the best of our knowledge, the first dataset investigating the effect of sex and age across the lifespan on the combination of self-administration and handling induced convulsions. We found that age of the animal significantly impacted the onset, but not magnitude, of HIC and that the difference in HIC onset was likely due to differences in alcohol metabolism. In addition, we found that aged animals drink significantly less than younger animals in a short term DID procedure but the lower alcohol consumption in aged animals still produced similar blood alcohol concentrations likely due, once again, to differences in alcohol metabolism.

There has long been an established negative correlation between the magnitude of HIC and alcohol self-administration (Crabbe et al., 1990; Metten et al., 1998; Ford et al., 2011). The current project demonstrates that in aged animals utilizing an often-used mouse strain, a similar relationship exists, specifically for baseline (pre-alcohol) handling induced convulsion scores. In the current project, baseline HIC scores were significantly greater in aged animals compared to younger animals and aged animals consumed significantly less alcohol in the DID procedure compared to younger animals. The significant increase in baseline HIC scores as a function of age suggesting increased central nervous system (CNS) hyperexcitability in aged animals compared to younger animals. It has been previously demonstrated that normal aging or early stages of Alzheimer’s disease can result in increased CNS hyperexcitability (Simkin et al., 2015; Aleogho et al., 2025; for review see Delbono et al., 2026) potentially impacting cognition in aged animals (Ho et al., 2022; reviewed in Foster, 2023) and increases in anxiety in aged animals (Matthews et al., 2024). In addition, aging can result in increased neuronal hyperexcitability in motor neurons (Delbono et al., 2026) which can produce involuntary muscle contractions (Hurst and Hobson-Webb, 2016) that may contribute to the increased HIC score in aged B6 mice in the current project. The inverse relationship we report in the present work between the increased baseline HIC score in aged subjects and the decreased alcohol self-administration in the same animals suggests it is likely that another complex behavior, namely voluntary alcohol self-administration, is impacted by CNS hyperexcitability that occurs as a function of aging.

Given the previously established inverse relationship between total HIC score and voluntary alcohol self-administration in mouse models (Metten and Crabbe, 2005) it was surprising that no significant difference was found in total HIC as a function of age given aged animals consumed less alcohol in the 4-day DID session. However, examination of Figure 1B reveals that total HIC is greater in aged animals compared to younger animals for both female and males even though a significant effect was not found. In addition, the associated figures in Figure 2 reveal that HIC develops later in aged animals following the 4.0 g/kg alcohol injection compared to younger animals. It is therefore likely that had we continued to collect HIC data past the 10-h post alcohol time point we would have found increased HIC scores in aged animals. We are currently exploring this hypothesis.

The later development of HIC in aged animals compared to adolescent and young adult animals is likely due to differences in alcohol metabolism. In support of this we determined blood alcohol concentrations in a novel set of aged, young adult and adolescent B6 mice 4-h following a 4.0 g/kg alcohol injection and found significantly higher BAC levels in aged animals. While we and others have demonstrated that similar BAC levels are found in aged rodents compared to younger animals if the dose of alcohol used is under ~2.0 g/kg alcohol and blood samples are taken within 90 min of the alcohol administration (Novier et al., 2013; Ornelas et al., 2015; Gano et al., 2017; Perkins et al., 2018; Matthews and Mittleman, 2017; Matthews et al., 2020; Matthews et al., 2024), it is known that aged animals metabolize alcohol slower over longer sample periods (Seitz et al., 1989; Watson et al., 2020). The slower alcohol metabolize in aged subjects agrees with human data from older adults (e.g., Schmucker and Wang, 1980; Seitz and Stickel, 2007; reviewed in Meier and Seitz, 2008) suggesting altered liver function in aged B6 animals is delaying the onset of acute alcohol withdrawal.

The differential blood alcohol concentration data following the 4.0 g/kg alcohol challenge raises several important concerns regarding alcohol research in aged animals. For example, research studies that employ chronic alcohol exposure in aged animals compared to younger animals (Matthews et al., 2019; Ho et al., 2022) run the risk of inadvertently confounding data sets due to longer and higher alcohol levels in aged animals compared to younger animals. While potentially problematic, there are research approaches that can overcome this obstacle. First, use of alcohol vapor chambers where alcohol flow rate can be modified by age to control for blood alcohol levels can be employed. However, alcohol vapor exposure lacks face validity. A second option is a systematic research endeavor to determination the blood alcohol levels over time as a function of age following both gavage and intraperitoneal alcohol exposures. While these critical BAC curves will likely vary due to age, sex, species and strain, the resultant data will prove invaluable to the further success of alcohol and aging research.

Aged B6 animals consumed significantly less alcohol in the 4-day drinking in the dark procedure, an effect that is similar to what was previously reported (Jimenez Chavez et al., 2022). We believe this is one of the few studies to investigate alcohol self-administration in aged female and male animals compared to younger animals. Recent studies have revealed that alcohol self-administration rates are increasing in the older demographic in many countries (Keyes, 2023; White et al., 2023) necessitating the investigation of alcohol self-administration as a function of age and sex. Due to the importance of determining BAC levels in animals that self-administer (Grahame, 2026) we measured BAC in a small subset of animals immediately following completion of the DID procedure and found similar blood alcohol concentrations across the three ages even though different self-administration rates existed across the three ages. The similar BAC levels even though different self-administration levels exist is likely impacted by alcohol metabolism. In addition, it is interesting to speculate that similar alcohol-induced impairments in motor function and/or cognitive function would be found in aged animals compared to younger animals due to the similar BAC even though self-administration rates were lower in the aged animals highlighting the potentially dangerous results of alcohol consumption in the older population. A Previous study (Jimenez Chavez et al., 2022) provides data supporting future work in this area.

There are experimental factors that should be improved upon in future research investigating acute alcohol withdrawal and alcohol self-administration in aged animals. First, the determination of BAC following alcohol self-administration in the DID study was statistical underpowered to query sex effects and consequently we collapsed across sex to investigate if BAC was similar, or different, across ages. Future studies should include enough subjects to also statistically investigate sex-dependent effects of BAC after DID in mice of different ages. Second, most studies utilizing the DID procedure place alcohol bottles on the cages 3-h following the beginning of the dark cycle while we placed the alcohol bottle on the cages 2-h following the beginning of the dark cycle. Previous research has shown that greater alcohol self-administration is associated with using the 3-h instead of the 2-h or a 1-h time point (Rhodes et al., 2005). However, considering this is the first study using aged animals in the DID procedure we were unsure if we would get greater, less, or the same level, of alcohol consumption in the aged animals so we selected the 2-h time point so our data would not be limited by either a ceiling or floor effect. Future research should use the 3-h time point to see if alcohol drinking continues to escalate in aged animals. Finally, HIC scoring should be extended beyond 10 h to ascertain if aged animals continue to have elevated HIC scores and a greater overall acute withdrawal effect following an alcohol challenge.

In conclusion, aged animals demonstrated a temporally altered acute withdrawal score as measured by handling induced convulsions and demonstrated reduced alcohol self-administration, particularly in aged females, using a short-term drinking in the dark procedure. Changes in alcohol metabolism due to aging were also identified suggesting multiple bodily systems ranging from liver to brain may be altered due to aging and impact the effect of alcohol. Additional research is needed to fully understand what biological systems are altered and how they impact intoxication in aged animals compared to younger animals.

## Data Availability

The raw data supporting the conclusions of this article will be made available by the authors, without undue reservation.

## References

[ref1] Aleogho B. M. Mohri M. Jang M. S. Tsukada S. Al-Hebri Y. Matsuyama H. J. . (2025). Aberrant neuronal hyperactivation causes an age-dependent behavioral decline in *Caenorhabditis elegans*. Proc. Natl. Acad. Sci. USA 122:e2412391122. doi: 10.1073/pnas.2412391122, 39739791 PMC11725918

[ref2] Anton P. E. Maphis N. M. Linsenbardt D. N. Coleman L. G. Jr. (2025). Excessive alcohol use as a risk factor for Alzheimer's disease: epidemiological and preclinical evidence. Adv. Exp. Med. Biol. 1473, 211–242. doi: 10.1007/978-3-031-81908-7_10, 40128481 PMC12720481

[ref3] Anton P. E. Rutt L. N. Kaufman M. L. Busquet N. Kovacs E. J. McCullough R. L. (2024). Binge ethanol exposure in advanced age elevates neuroinflammation and early indicators of neurodegeneration and cognitive impairment in female mice. Brain Behav. Immun. 116, 303–316. doi: 10.1016/j.bbi.2023.12.034, 38151165 PMC11446185

[ref4] Bauer M. R. McVey M. M. Germano D. M. Zhang Y. Boehm S. L. 2nd (2022). Intra-dorsolateral striatal AMPA receptor antagonism reduces binge-like alcohol drinking in male and female C57BL/6J mice. Behav. Brain Res. 418:113631. doi: 10.1016/j.bbr.2021.113631, 34715146 PMC8671209

[ref5] Buck K. J. Metten P. Belknap J. K. Crabbe J. C. (1997). Quantitative trait loci involved in genetic predisposition to acute alcohol withdrawal in mice. J. Neurosci. 17, 3946–3955. doi: 10.1523/JNEUROSCI.17-10-03946.1997, 9133412 PMC6573712

[ref6] Calvo E. Medina J. T. Ornstein K. A. Staudinger U. M. Fried L. P. Keyes K. M. (2020). Cross-country and historical variation in alcohol consumption among older men and women: leveraging recently harmonized survey data in 21 countries. Drug Alcohol Depend. 215:108219. doi: 10.1016/j.drugalcdep.2020.108219, 32795884 PMC7585691

[ref7] Chang J. S. Huang H. Z. Yuan M. Zhou Y. Liu D. Zhan K. B. . (2025). Alcohol addiction and Alzheimer's disease: a molecular collision course. Transl. Psychiatry 15:410. doi: 10.1038/s41398-025-03619-6, 41107250 PMC12534461

[ref8] Crabbe J. C. Feller D. J. Terdal E. S. Merrill C. D. (1990). Genetic components of ethanol responses. Alcohol 7, 245–248. doi: 10.1016/0741-8329(90)90013-3, 2184836

[ref9] DelbonoO. WangZ. M. MessiM. L. (2026). The rise and deceleration of neuronal excitability in aging and Alzheimer's disease: mechanisms, implications, and therapeutic targets. Ageing Res. Rev. 114:102999. doi: 10.1016/j.arr.2025.102999, 41429406

[ref10] Dorobisz K. Dorobisz T. Pazdro-Zastawny K. Janczak M. Czyż K. (2025). The impact of alcohol addiction on the quality of life, mental condition, clinical condition and nutritional status of patients with head and neck cancer. Alcohol 129, 150–156. doi: 10.1016/j.alcohol.2025.10.005, 41135818

[ref11] Esser M. B. Sherk A. Liu Y. Henley S. J. Naimi T. S. (2024). Reducing alcohol use to prevent cancer deaths: estimated effects among U.S. adults. Am. J. Prev. Med. 66, 725–729. doi: 10.1016/j.amepre.2023.12.003, 38514233 PMC10963036

[ref12] Faccidomo S. Eastman V. R. Santanam T. S. Swaim K. S. Taylor S. M. Hodge C. W. (2025). Distinct sex differences in ethanol consumption and operant self-administration in C57BL/6J mice with uniform regulation by glutamate AMPAR activity. Front. Behav. Neurosci. 18:1498201. doi: 10.3389/fnbeh.2024.1498201, 39911242 PMC11794300

[ref13] Ford M. M. Fretwell A. M. Anacker A. M. Crabbe J. C. Mark G. P. Finn D. A. (2011). The influence of selection for ethanol withdrawal severity on traits associated with ethanol self-administration and reinforcement. Alcohol. Clin. Exp. Res. 35, 326–337. doi: 10.1111/j.1530-0277.2010.01348.x, 21070250 PMC3058687

[ref14] Foster T. C. (2023). Animal models for studies of alcohol effects on the trajectory of age-related cognitive decline. Alcohol 107, 4–11. doi: 10.1016/j.alcohol.2022.04.005, 35504438

[ref15] Gano A. Doremus-Fitzwater T. L. Deak T. (2017). A cross-sectional comparison of ethanol-related cytokine expression in the hippocampus of young and aged Fischer 344 rats. Neurobiol. Aging 54, 40–53. doi: 10.1016/j.neurobiolaging.2017.01.025, 28319836 PMC5401774

[ref16] Grahame N. J. (2026). Argument for the pharmacokinetically-informed preclinical researcher: a commentary for alcohol. Alcohol 130, 1–5. doi: 10.1016/j.alcohol.2025.11.003, 41260317 PMC13543843

[ref17] Ho A. M. Peyton M. P. Scaletty S. J. Trapp S. Schreiber A. Madden B. J. . (2022). Chronic intermittent ethanol exposure alters behavioral flexibility in aged rats compared to adult rats and modifies protein and protein pathways related to Alzheimer's disease. ACS Omega 7, 46260–46276. doi: 10.1021/acsomega.2c04528, 36570296 PMC9774340

[ref18] Hurst R. L. Hobson-Webb L. D. (2016). Therapeutic implications of peripheral nerve Hyperexcitability in muscle cramping: a retrospective review. J. Clin. Neurophysiol. 33, 560–563. doi: 10.1097/WNP.0000000000000291, 27258601

[ref19] Islami F. Marlow E. C. Thomson B. McCullough M. L. Rumgay H. Gapstur S. M. . (2024). Proportion and number of cancer cases and deaths attributable to potentially modifiable risk factors in the United States, 2019. CA Cancer J. Clin. 74, 405–432. doi: 10.3322/caac.21858, 38990124

[ref20] Jimenez Chavez C. L. Van Doren E. Matalon J. Ogele N. Kharwa A. Madory L. . (2022). Alcohol-drinking under limited-access procedures during mature adulthood accelerates the onset of cognitive impairment in mice. Front. Behav. Neurosci. 16:732375. doi: 10.3389/fnbeh.2022.732375, 35685271 PMC9171112

[ref21] Jones C. H. Dolsten M. (2024). Healthcare on the brink: navigating the challenges of an aging society in the United States. NPJ Aging 10:22. doi: 10.1038/s41514-024-00148-2, 38582901 PMC10998868

[ref22] Kerr E. L. Garscia A. Hartwig J. Stumo S. Crippen A. Sharma P. . (2025). Alcohol produces sex differences in ataxia but not nonspatial cognition in aged rats compared to younger rats. Alcohol Alcohol. 60:agaf049. doi: 10.1093/alcalc/agaf049, 40755268

[ref23] Keyes K. M. (2023). Alcohol use in the older adult US population: trends, causes, and consequences. Alcohol 107, 28–31. doi: 10.1016/j.alcohol.2022.05.005, 35661693

[ref24] Lassman D. Hartman M. Washington B. Andrews K. Catlin A. (2014). US health spending trends by age and gender: selected years 2002-10. Health Aff. (Millwood). 33, 815–822. doi: 10.1377/hlthaff.2013.1224, 24799579

[ref25] Matthews D. B. Koob G. F. (2023). On the critical need to investigate the effect of alcohol in the older population. Alcohol 107, 2–3. doi: 10.1016/j.alcohol.2022.09.002, 36202275

[ref26] Matthews D. B. Mittleman G. (2017). Age-dependent effects of chronic intermittent ethanol treatment: gross motor behavior and body weight in aged, adult and adolescent rats. Neurosci. Lett. 657, 146–150. doi: 10.1016/j.neulet.2017.08.012, 28789984

[ref27] Matthews D. B. Rossmann G. (2023). Using animal models to identify clinical risk factors in the older population due to alcohol use and misuse. Alcohol 107, 38–43. doi: 10.1016/j.alcohol.2022.05.003, 35659578

[ref28] Matthews D. B. Rossmann G. Matthews S. J. Zank A. Shult C. Turunen A. . (2024). Increased anxiolytic effect in aged female rats and increased motoric behavior in aged male rats to acute alcohol administration: comparison to younger animals. Pharmacol. Biochem. Behav. 239:173770. doi: 10.1016/j.pbb.2024.173770, 38636813

[ref29] Matthews D. B. Scaletty S. Schreiber A. Trapp S. (2020). Acute ethanol administration produces larger spatial and nonspatial memory impairments in 29-33 month old rats compared to adult and 18-24 month old rats. Pharmacol. Biochem. Behav. 199:173074. doi: 10.1016/j.pbb.2020.173074, 33212145

[ref30] Matthews D. B. Watson M. R. James K. Kastner A. Schneider A. Mittleman G. (2019). The impact of low to moderate chronic intermittent ethanol exposure on behavioral endpoints in aged, adult, and adolescent rats. Alcohol 78, 33–42. doi: 10.1016/j.alcohol.2018.11.005, 30472308

[ref31] McKee S. A. Earnshaw V. A. Epstein E. Hughes T. L. Johnston A. D. Lee C. S. . (2025). Women and alcohol: a call to action. Alcohol. Clin. Exp. Res. 50:205. doi: 10.1111/acer.70205, 41287268 PMC13202574

[ref32] McKetta S. C. Keyes K. M. (2020). Trends in U.S. women's binge drinking in middle adulthood by socioeconomic status, 2006-2018. Drug Alcohol Depend. 212:108026. doi: 10.1016/j.drugalcdep.2020.108026, 32408139 PMC7293936

[ref33] Meier P. Seitz H. K. (2008). Age, alcohol metabolism and liver disease. Curr. Opin. Clin. Nutr. Metab. Care 11, 21–26. doi: 10.1097/MCO.0b013e3282f30564, 18090653

[ref34] Metten P. Crabbe J. C. (2005). Alcohol withdrawal severity in inbred mouse (*Mus musculus*) strains. Behav. Neurosci. 119, 911–925. doi: 10.1037/0735-7044.119.4.911, 16187819

[ref35] Metten P. Phillips T. J. Crabbe J. C. Tarantino L. M. McClearn G. E. Plomin R. . (1998). High genetic susceptibility to ethanol withdrawal predicts low ethanol consumption. Mamm. Genome 9, 983–990. doi: 10.1007/s003359900911. 9880664, 9880664

[ref36] Novier A. Van Skike C. E. Diaz-Granados J. L. Mittleman G. Matthews D. B. (2013). Acute alcohol produces ataxia and cognitive impairments in aged animals: a comparison between young adult and aged rats. Alcohol. Clin. Exp. Res. 37, 1317–1324. doi: 10.1111/acer.12110, 23550918

[ref37] Ornelas L. C. Novier A. Van Skike C. E. Diaz-Granados J. L. Matthews D. B. (2015). The effects of acute alcohol on motor impairments in adolescent, adult, and aged rats. Alcohol 49, 121–126. doi: 10.1016/j.alcohol.2014.12.002, 25613215

[ref38] Perkins A. E. Vore A. S. Lovelock D. Varlinskaya E. Deak T. (2018). Late aging alters behavioral sensitivity to ethanol in a sex-specific manner in Fischer 344 rats. Pharmacol. Biochem. Behav. 175, 1–9. doi: 10.1016/j.pbb.2018.07.012, 30171932 PMC6240477

[ref39] Philip V. M. Duvvuru S. Gomero B. Ansah T. A. Blaha C. D. Cook M. N. . (2010). High-throughput behavioral phenotyping in the expanded panel of BXD recombinant inbred strains. Genes Brain Behav. 9, 129–159. doi: 10.1111/j.1601-183X.2009.00540.x, 19958391 PMC2855868

[ref40] Radke A. K. Sneddon E. A. Frasier R. M. Hopf F. W. (2021). Recent perspectives on sex differences in compulsion-like and binge alcohol drinking. Int. J. Mol. Sci. 22:3788. doi: 10.3390/ijms22073788, 33917517 PMC8038761

[ref41] Rhodes J. S. Best K. Belknap J. K. Finn D. A. Crabbe J. C. (2005). Evaluation of a simple model of ethanol drinking to intoxication in C57BL/6J mice. Physiol. Behav. 84, 53–63. doi: 10.1016/j.physbeh.2004.10.007, 15642607

[ref42] Schmucker D. L. Wang R. K. (1980). Age-related changes in liver drug-metabolizing enzymes. Exp. Gerontol. 15, 423–431. doi: 10.1016/0531-5565(80)90050-9, 6775972

[ref43] Seitz H. K. Meydani M. Ferschke I. Simanowski U. A. Boesche J. Bogusz M. . (1989). Effect of aging on in vivo and in vitro ethanol metabolism and its toxicity in F344 rats. Gastroenterology 97, 446–456. doi: 10.1016/0016-5085(89)90082-6, 2744358

[ref44] Seitz H. K. Stickel F. (2007). Alcoholic liver disease in the elderly. Clin. Geriatr. Med. 23, 905–921. doi: 10.1016/j.cger.2007.06.010, 17923345

[ref45] Simkin D. Hattori S. Ybarra N. Musial T. F. Buss E. W. Richter H. . (2015). Aging-related Hyperexcitability in CA3 pyramidal neurons is mediated by enhanced A-type K+ channel function and expression. J. Neurosci. 35, 13206–13218. doi: 10.1523/JNEUROSCI.0193-15.2015, 26400949 PMC4579378

[ref46] Tampi R. R. Tampi D. J. Durning M. (2015). Substance use disorders in late life: a review of current evidence. Healthy Aging Res. 4:27. doi: 10.12715/har.2015.4.27

[ref47] Tampi R. R. Tampi D. J. Elson A. (2022). Substance use disorders in the elderly. Psychiatr. Clin. North Am. 45, 707–716. doi: 10.1016/j.psc.2022.07.005, 36396274

[ref48] The United States Census Bureau, (2025) Available online at: https://www.census.gov/programs-surveys/acs/news/updates/2025.html (Accessed December 15, 2025).

[ref49] Thiele T. E. Navarro M. (2014). "Drinking in the dark" (DID) procedures: a model of binge-like ethanol drinking in non-dependent mice. Alcohol 48, 235–241. doi: 10.1016/j.alcohol.2013.08.005, 24275142 PMC4004717

[ref50] Van Skike C. E. Botta P. Chin V. S. Tokunaga S. McDaniel J. M. Venard J. . (2010). Behavioral effects of ethanol in cerebellum are age dependent: potential system and molecular mechanisms. Alcohol. Clin. Exp. Res. 34, 2070–2080. doi: 10.1111/j.1530-0277.2010.01303.x, 20860615 PMC2988915

[ref51] Watson M. R. James K. Mittleman G. Matthews D. B. (2020). Impact of acute ethanol exposure on body temperatures in aged, adult and adolescent male rats. Alcohol 82, 81–89. doi: 10.1016/j.alcohol.2019.08.001, 31408671

[ref52] White A. M. (2020). Gender differences in the epidemiology of alcohol use and related harms in the United States. Alcohol Res. 40:01. doi: 10.35946/arcr.v40.2.01, 33133878 PMC7590834

[ref53] White A. M. Orosz A. Powell P. A. Koob G. F. (2023). Alcohol and aging - an area of increasing concern. Alcohol 107, 19–27. doi: 10.1016/j.alcohol.2022.07.005, 35940508

[ref54] Witkiewitz K. Leggio L. (2025). Sex and gender differences in alcohol use disorder: quo Vadis? Alcohol 123, 121–125. doi: 10.1016/j.alcohol.2025.01.003, 39837376

[ref55] World Health Organization. Ageing and health (2025). Available online at: https://www.who.int/news-room/fact-sheets/detail/ageing-and-health (Accessed December 15, 2025).

[ref56] Yoon H. J. Doyle M. A. Altemus M. E. Bethi R. Lago S. H. Winder D. G. . (2025). Operant ethanol self-administration behaviors do not predict sex differences in continuous access home cage drinking. Alcohol 123, 87–99. doi: 10.1016/j.alcohol.2024.08.004, 39218047 PMC12034132

[ref57] Zahr N. M. (2024). Alcohol use disorder and dementia: a review. Alcohol Res. 44:03. doi: 10.35946/arcr.v44.1.03, 38812709 PMC11135165

[ref58] Zirulnik A. Liu S. Wells M. Alter S. M. Engstrom G. Solano J. J. . (2024). Alcohol use is associated with intracranial hemorrhage in older emergency department head trauma patients. J Am Coll Emerg Physicians Open. 5:e13245. doi: 10.1002/emp2.13245, 39086794 PMC11289673

